# Resonator-free optical bistability based on epsilon-near-zero mode

**DOI:** 10.1038/s41598-019-43067-z

**Published:** 2019-04-25

**Authors:** Myunghwan Kim, Sangin Kim, Soeun Kim

**Affiliations:** 1 Integrated Optics Laboratory, Advanced Photonics Research Institute, GIST, Gwangju, 61005 South Korea; 2 Department of Electrical and Computer Engineering, Ajou University, Suwon, 16499 South Korea

**Keywords:** Nonlinear optics, Integrated optics

## Abstract

A majority of existing research on optical bistability rely on resonant schemes using nonlinear materials, which inevitably require a trade-off between the switching intensity and response time. In this work, we propose a novel non-resonant scheme, which utilizes strong light enhancement of the epsilon-near-zero (ENZ) mode to realize optical bistability. We used graphene as a non-linear ENZ material and designed an integrated optical bistability device composed of a graphene-embedded Si waveguide, which supports an ENZ mode. The proposed scheme can overcome the trade-off necessary in resonance-based optical bistability, and the designed optical bistability device simultaneously allows for a short response time (~200 fs) and low switching intensity (~700 kW/cm^2^).

## Introduction

Optical bistability is one of the key functionalities required to implement all-optical switching and optical memory that are essential functions for all-optical signal processing^1–3^. The optical bistability is a nonlinear phenomenon that has two stable output states for a given input state and exhibits hysteresis; the steady-state output depends on the history of the excitation. This phenomenon can be induced by a bell-shaped output response to an input variation, which is generally caused by a feed-back mechanism in a resonator with nonlinear refractive index or absorption. For practical applications of the optical bistability, it is important to realize a low switching intensity and short response time simultaneously. So far, numerous studies have focused on reducing the switching intensity, and various resonance-based schemes have been proposed to reduce the switching intensity with high quality factor (Q) and strong field enhancement, such as photonic crystals^4,5^, surface plasmons^6^, ring resonators^7,8^, and waveguide gratings with guided mode resonance^9^. However, because the response time is proportional to the Q factor, reducing switching intensity by increasing Q factor inevitably increases the response time^9^. Therefore, these resonant schemes cannot avoid a trade-off between a response time and a switching intensity.

To avoid this trade-off, non-resonant schemes for optical bistability have been proposed, which achieve fast optical bistability by effective permittivity variation^10^ and the hyperbolic dispersion property^11^ of the metal-dielectric structure, resulting in response times of ~1 ps and ~350 fs, respectively. However, the switching intensity is above 100 MW/cm^2^, which should be reduced.

In this study, a simple integrated waveguide structure supporting the epsilon-near-zero (ENZ) mode is proposed to realize optical bistability at a wavelength of 1.55 *µ*m. The ENZ mode defined as an even-symmetry plasmonic mode (long-range surface plasmonic mode) in ultra-thin ENZ material (|ε| = ~0) confines most of the electric field in the ultra-thin film^12–14^. We realized optical bistability with strong non-resonant light confinement of the ENZ mode in a nonlinear ENZ material. We used monolayer graphene as a nonlinear ENZ material. Graphene is widely used as an active nonlinear material for optical bistability because of its tunable permittivity and high nonlinear coefficient^15–18^. We show that graphene supports the ENZ mode and the nonlinearity of graphene combined with the ENZ mode effect results in optical bistability, simultaneously achieving a short response time and low switching intensity. All calculations in this paper have been carried out using finite-difference time-domain (FDTD) method.

## Results

### Epsilon near zero mode in grapheme

**Figure 1:**
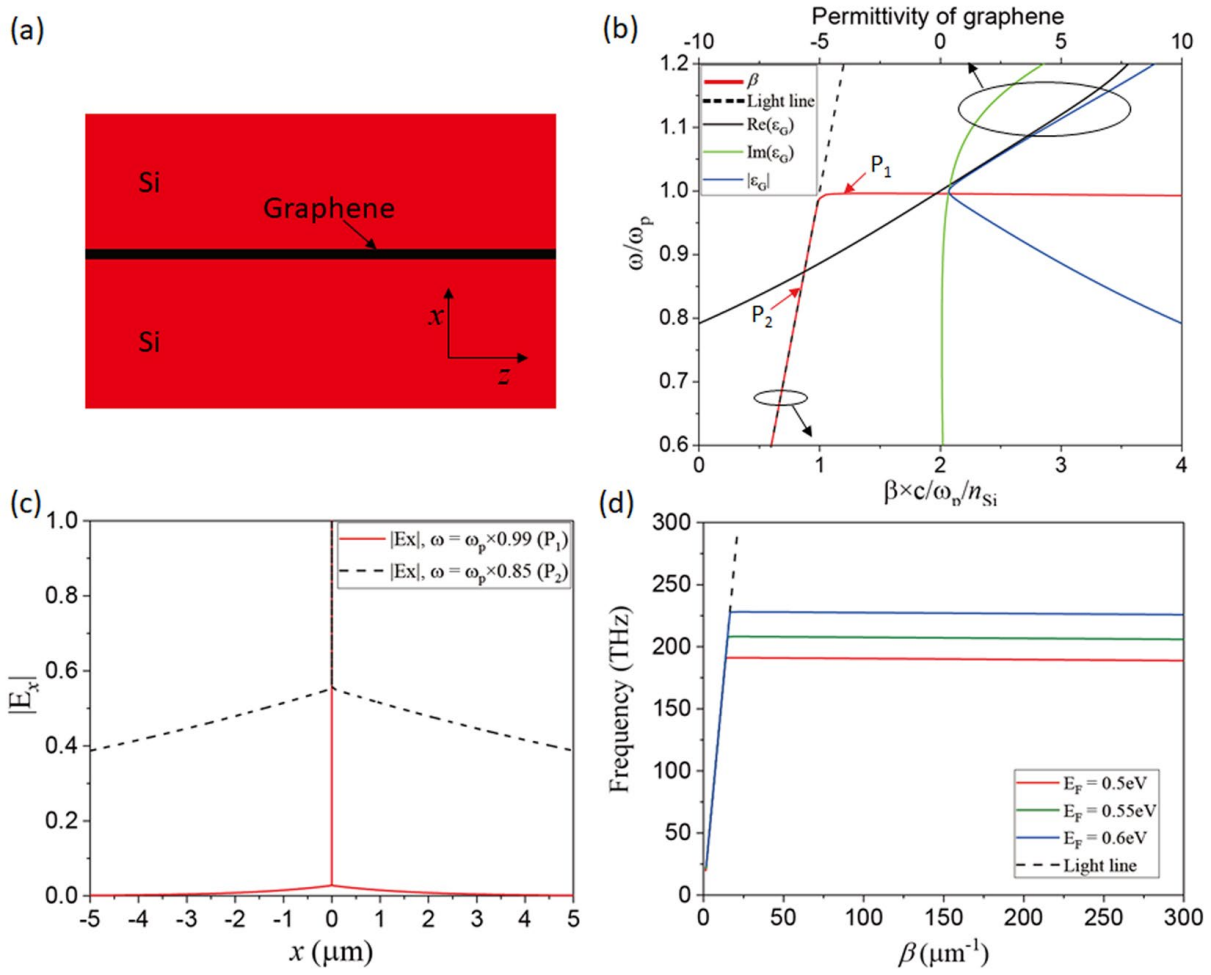
(**a**) Three-layer Si-graphene-Si structure. (**b**) Dispersion curve of guided mode in Si/Graphene/Si geometry shown in (**a**) and the complex permittivity of graphene for Fermi level of E_F_ = 0.5 eV. (**c**) Amplitude of the surface-normal component of the electric field |E_*x*_| for E_F_ = 0.5 eV at P_1_ (red line) and P_2_ (black dash) indicted in (**b**). (**d**) Dispersion curves of Si/Graphene/Si geometry for various Fermi level: E_F_ = 0.5 eV.

For the investigation of the ENZ mode in graphene, a Si/graphene/Si structure as shown in Fig. 1(a) is considered. The conductivity of graphene is obtained by Kubo formalisms^19,20^ assuming a Fermi velocity of ν_F_ = 10^6^ *m/s* and a mobility of *μ* = 10,000 *cm*^2^/Vs. The permittivity of graphene is obtained from ε_G_ = 1 + iσ/(ωε_0_d_G_), assuming the thickness of monolayer graphene, d_G_ = 0.34 nm. The permittivity of Si is set as 3.45. Figure 1(b) shows the real part, imaginary part, and magnitude of permittivity of graphene for Fermi level of E_F_ = 0.5 eV as a function of frequency. It can be seen that the real part of the permittivity of graphene is zero and the magnitude of graphene permittivity is minimized at the plasma frequency (ω_p_). We also plot a dispersion curve of the guided mode in Si/graphene/Si structure for E_F_ = 0.5 eV in Fig. 1(b). The implicit dispersion equation (ω - β relation) for transverse magnetic (TM) polarization was derived from Maxwell’s equations by satisfying the boundary conditions, and it was solved numerically; for a given real-valued transverse wave-number (β), a corresponding complex-valued frequency (ω) was calculated using the root-finding algorithm. Note that, only the part of the dispersion curves corresponding to the guided mode, which lies under the light line (black dashed line), is plotted here. In the frequency region far below the plasma frequency of graphene, the dispersion curve is very close to the light line, which is similar to even-symmetry plasmonic modes (long-range surface plasmonic modes) in a thin metallic film. However, a typical characteristic of the ENZ mode is shown when the dispersion curve rapidly becomes flat near the plasma frequency^12^. Figure 1(c) shows the amplitude profiles of the surface-normal component of the electric field at two different frequency regions: one in the ENZ mode region (denoted as P_1_ (ω = 0.99ω_p_) in Fig. 1(b)) and the other in the non-ENZ mode regions (denoted as P_2_ (ω = 0.85ω_p_) in Fig. 1(b)). At P_1_, most of the electric field is confined to the graphene layer, resulting in large optical absorption although Im(ε_G_) is very small. Contrastingly, at P_2_, the absorption is negligibly small because the electric field spreads into the Si region. Figure 1(d) shows the dispersion curves for the different Fermi levels. Because the graphene plasma frequency varies with the Fermi level, the ENZ mode region shifts correspondingly. Therefore, the mode property at a fixed frequency can be varied remarkably by changing the Fermi level of graphene.

### Optical bistability based on ENZ

**Figure 2:**
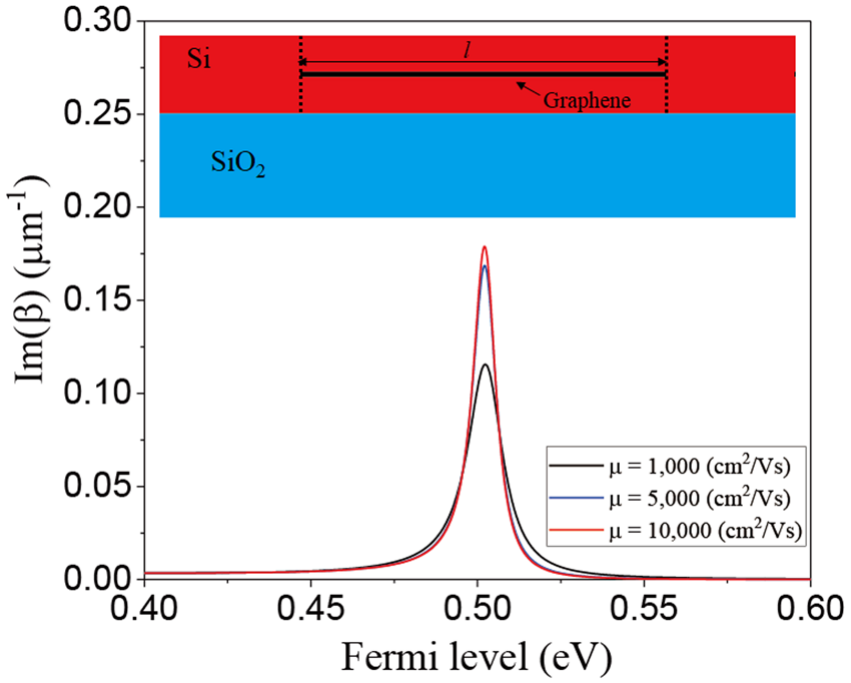
Imaginary part of the wave-number in the proposed optical bistability device as a function of the Fermi level (E_F_) for different mobility values (*µ*). Inset shows the schematic of the proposed optical bistability device.

The proposed structure to realize optical bistability is schematically illustrated in the inset of Fig. 2, which consists of a Si waveguide and a graphene sheet embedded in the Si layer, where the thicknesses of the graphene sheet and Si waveguide are assumed to be 0.34 nm and 220 nm, respectively. Figure 2 shows the imaginary part of the wave-number at λ = 1.55 *µ*m as a function of the Fermi level of graphene for different mobility values, which represents the loss in the guided mode. It can be seen that the imaginary part of the wave-number increases when the Fermi level approaches E_F_ = 0.5 eV, where the permittivity of graphene is almost zero at λ = 1.55 *µ*m. As the Fermi level approaches the ENZ region (E_F_ = 0.5 eV), the electric field becomes confined to the graphene layer owing to the ENZ mode, which results in an increase in the loss in the graphene. In addition, the loss in the guided mode can be tuned by adjusting the Fermi level at fixed wavelength.

We exploited the strong and tunable field confinement and nonlinearity of graphene to realize optical bistability. Because the permittivity of graphene can be modified through the nonlinear Kerr effect, the same effect as the Fermi level shift can be implemented by varying the input intensity. In other words, by modifying the input intensity, the light confinement can be tuned. Therefore, the loss in the mode can be tuned to a considerable extent by adjusting the input intensity.

The imaginary part of wave number (i.e., loss) varies with respect to the variation in mobility, and its slope and peak value deteriorate as the mobility decreases. This stems from the degradation of the ENZ effect due to poor graphene quality. It appears that a graphene mobility of *µ* = 5,000 cm^2^/Vs is sufficient for obtaining high performance optical bistability because the imaginary part of wave-number does not vary much for graphene mobility values larger than *µ* = 5,000 cm^2^/Vs.

**Figure 3:**
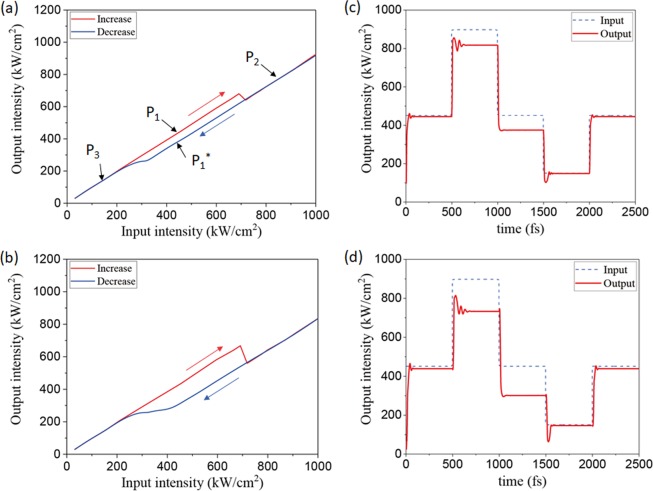
Optical bistability curves and temporal response based on ENZ mode for various device length (l). (**a**,**b**) optical bistability curves for *l* = 500 nm and *l* = 1 µm, respectively. (**c**,**d**) temporal response of bistability for *l* = 500 nm and *l* = 1 µm, respectively. The blue dot denotes input intensity with the variation of P_1_ → P_2_→ P_1_* → P_3_ → P_1_ indicated in (**a**), and the red line denotes corresponding output intensity.

We set the operating Fermi level to E_F_ = 0.515 eV, which deviates slightly from the ENZ region, and in this case, the permittivity of graphene is ε_G_ = −1.07527 + 0.217745*i* at λ = 1.55 *µ*m. The nonlinear susceptibility of graphene is assumed to be 2.095 × 10^−15^ ^21,22^. Because the non-ENZ mode is supported for the operational Fermi level (E_F_ = 0.515 eV) and low input intensity, the loss associated with the mode is very small. By contrast, as the input intensity increases, the permittivity of graphene approaches the ENZ region and the guided mode changes to the ENZ mode. Consequently, the loss associated with the mode rapidly increases because of the enhanced electric field confinement in graphene. However, if the input intensity increases further, the permittivity of graphene drifts away from the ENZ region and thus, the loss decreases again as a result of the weakened confinement of the non-ENZ mode. This functions as a feedback mechanism for optical bistability, which shows features similar to the bell-shaped transmission-input power relationship in resonator-based optical bistable devices^23^. Figures 3(a,b) show optical bistability behavior for various lengths (*l*) of the proposed structure: (a) *l* = 500 nm and (b) *l* = 1 *µ*m. The red and blue curves represent the output intensities for the increasing and decreasing input intensities, respectively. The calculated switching intensities are the same as 700 kW/cm^2^ regardless of the device length. However, the hysteresis width decreases as the device length decreases because the propagation length in the ENZ mode is shorter, which results in the weaker ENZ mode effect. Figure 3(c,d) show the temporal responses of the optical bistability for various device lengths: (c) *l* = 500 nm and (d) *l* = 1 *µ*m. To analyze the temporal response, we first set the input intensity below the switching intensity (denoted as P_1_) for 500 fs and applied an additional 500-fs wide square pulse of 450 kW/cm^2^ height to the input, triggering the output transition from P_1_ to P_1_* through P_2_. Then, we triggered the output transition from P_1_* to P_1_ through P_3_ by applying another 500-fs wide square pulse of −300 kW/cm^2^ height to the input. As seen in Fig. 3(c,d), the output intensity shows overshooting and oscillating behavior before stabilization only when it goes through a nonlinear variation, that is, in the processes of P_1_ → P_2_ and P_1_* → P_3_. The amplitude of the overshooting variation appears to be nearly the same as the abrupt variation in output intensity in each nonlinear process. Note that for the transitions of P_2_ → P_1_* and P_3_ → P_1_, the overshooting and oscillating behavior is not observed where there is no nonlinear variation of the output. In nonlinear processes, the abrupt change in intensity inside the device will cause abrupt changes in the local guided mode profile, and mode mismatch occurs in addition to the change in propagation loss. In general, at the mode mismatch, the incident wave requires a certain propagation distance to adapt to the mode. During the mode adaptation, the local propagation loss will also change, resulting in another change in the local mode. Therefore, this interaction between the change in mode profile and the propagation loss at the local mode mismatch seems to cause the oscillation behavior, which takes some time for stabilization. By contrast, in the linear processes, the gradual variation in the loss causes no abrupt local mode mismatch and thus, no oscillating behavior. The estimated rising and falling response times for the nonlinear transitions are about 200 fs and 150 fs, respectively. In the proposed structure, optical bistability is realized based on the guided-mode confinement variation near the ENZ mode, which is the non-resonant scheme. Therefore, the buildup time for the change in refractive index of graphene to support the ENZ mode is very short, and fast optical bistability behavior could be achieved. It should be noted that the device length does not affect the response time considerably because the propagation time of the guided mode for a few hundred nanometers is just a few femtoseconds. It appears that in the short device at the wavelength scale, the response time is mainly determined by the stabilizing time of the overshoot power.

## Conclusions

We numerically investigated the optical bistability behavior based on the ENZ mode in graphene. We showed that a simple, graphene-embedded Si waveguide structure supports an ENZ mode and its propagation loss varies drastically with the permittivity change in graphene, which enables optical bistability by virtue of the optical nonlinearity of graphene. The switching intensity and response time in the proposed structure are approximately 700 kW/cm^2^ and 200 fs, respectively. The proposed scheme may lead to a new class of non-resonant optically bistable devices that can avoid the hitherto necessary tradeoff between the switching intensity and response time. In addition, we believe that the proposed scheme can be applied to other material systems that support the ENZ mode such as an ultra-thin doped semiconductor layer, achieving similar optical bistability behavior.

## Methods

The dispersion relation of the guided mode was calculated by solving the one-dimensional Maxwell’s equation using the root-finding algorithm. In our numerical analysis of the optical bistability, the permittivity of graphene was calculated using the Kubo formula, assuming a graphene thickness of 0.34 nm, Fermi velocity of 10^6^ m/s, and mobility of 10,000 cm^2^/Vs, and the nonlinear susceptibility of graphene was assumed to be χ^(3)^ = 2.095 × 10^−15 ^^21,22^. The optical bistability curve and the temporal response were calculated using the commercial FDTD software (Lumerical FDTD). In the FDTD calculation, the third-order (Kerr-type) nonlinearity is handled by the relation of *E*(t) = *D*(t)/(ε_ο_ε_r_ + ε_ο_χ^(3)^|*E*(t-Δt)|^2^) assuming an instantaneous nonlinear response, where Δt is the time step of the iterative field update^24^. The latest value of *E* calculated from the latest value of *D* and the previous value of *E* is used for the update of *H* in the next iteration, which is followed by the update of *D*. This process repeats cyclically. The input and output intensities were calculated by integrating the Poynting vector over the waveguide cross section and calculating its time average.

## References

[1] Gibbs, H. M. *Optical Bistability: Controlling Light with Light*. (Academic, 1985).

[2] Mazurenko DA (2003). Ultrafast optical switching in three-dimensional photonic crystals. Phys. Rev. Lett..

[3] Nihei H, Okamoto A (2001). Switching time of optical memory devices composed of photonic crystals with an impurity three-level atom. Japanese J. Appl. Physics, Part 1 Regul. Pap. Short Notes Rev. Pap..

[4] Mingaleev SF, Kivshar YS (2002). Nonlinear transmission and light localization in photonic-crystal waveguides. J. Opt. Soc. Am. B.

[5] Soljačić M, Ibanescu M, Johnson SG, Fink Y, Joannopoulos JD (2002). Optimal bistable switching in nonlinear photonic crystals. Phys. Rev. E - Stat. Physics, Plasmas, Fluids, Relat. Interdiscip. Top..

[6] Prakash S, Das B, Venugopal R (2006). Optical bistability in nonlinear surface-plasmon polaritonic crystals. J. Sci. Ind. Res. (India)..

[7] Rukhlenko ID, Premaratne M, Agrawal GP (2010). Analytical study of optical bistability in Silicon Ring Resonators. Opt. Lett..

[8] Priem G (2005). Optical bistability and pulsating behaviour in Silicon-On-Insulator ring resonator structures. Opt. Express.

[9] Ngo QM, Kim S, Song SH, Magnusson R (2009). Optical bistable devices based on guided-mode resonance in slab waveguide gratings. Opt. Express.

[10] Husakou A, Herrmann J (2007). Steplike Transmission of Light through a Metal-Dielectric Multilayer Structure due to an Intensity-Dependent Sign of the Effective Dielectric Constant. Phys. Rev. Lett..

[11] Kim M, Kim S, Kim S (2018). Optical bistability based on hyperbolic metamaterials. Opt. Express.

[12] Campione S, Brener I, Marquier F (2015). Theory of epsilon-near-zero modes in ultrathin films. Phys. Rev. B - Condens. Matter Mater. Phys..

[13] Vassant S, Hugonin J-P, Marquier F, Greffet J-J (2012). Berreman mode and epsilon near zero mode. Opt. Express.

[14] Vassant S (2012). Epsilon-near-zero mode for active optoelectronic devices. Phys. Rev. Lett..

[15] Dai X, Jiang L, Xiang Y (2015). Low threshold optical bistability at terahertz frequencies with graphene surface plasmons. Sci. Rep..

[16] Guo J (2017). Low threshold optical bistability in one-dimensional gratings based on graphene plasmonics. Opt. Express.

[17] Ahn KJ, Rotermund F (2017). Terahertz optical bistability of graphene in thin layers of dielectrics. Opt. Express.

[18] Peres NMR, Bludov YV, Santos JE, Jauho AP, Vasilevskiy MI (2014). Optical bistability of graphene in the terahertz range. Phys. Rev. B - Condens. Matter Mater. Phys..

[19] Koppens FHL, Chang DE, García De Abajo FJ (2011). Graphene plasmonics: A platform for strong light-matter interactions. Nano Lett..

[20] Hanson GW (2008). Dyadic Green’s functions and guided surface waves for a surface conductivity model of graphene. J. Appl. Phys..

[21] Soh DBS, Hamerly R, Mabuchi H (2016). Comprehensive analysis of the optical Kerr coefficient of graphene. Phys. Rev. A.

[22] Hendry E, Hale PJ, Moger J, Savchenko AK, Mikhailov SA (2010). Coherent nonlinear optical response of graphene. Phys. Rev. Lett..

[23] Haus, H. A. *Waves and Field in Optoelectronics*. (Prentice Hall, 1983).

[24] Joseph RM, Taflove A (1997). FDTD Maxwell’s equations models for nonlinear electrodynamics and optics. IEEE Trans. Antennas Prop..

